# Reduced HGF/MET Signaling May Contribute to the Synaptic Pathology in an Alzheimer's Disease Mouse Model

**DOI:** 10.3389/fnagi.2022.954266

**Published:** 2022-07-12

**Authors:** Jing Wei, Xiaokuang Ma, Antoine Nehme, Yuehua Cui, Le Zhang, Shenfeng Qiu

**Affiliations:** Basic Medical Sciences, University of Arizona College of Medicine-Phoenix, Phoenix, AZ, United States

**Keywords:** Alzheimer's disease, MET receptor tyrosine kinase, hepatocyte growth factor, synaptic plasticity, regeneration

## Abstract

Alzheimer's disease (AD) is a neurodegenerative disorder strongly associates with aging. While amyloid plagues and neurofibrillary tangles are pathological hallmarks of AD, recent evidence suggests synaptic dysfunction and physical loss may be the key mechanisms that determine the clinical syndrome and dementia onset. Currently, no effective therapy prevents neuropathological changes and cognitive decline. Neurotrophic factors and their receptors represent novel therapeutic targets to treat AD and dementia. Recent clinical literature revealed that MET receptor tyrosine kinase protein is reduced in AD patient's brain. Activation of MET by its ligand hepatocyte growth factor (HGF) initiates pleiotropic signaling in the developing brain that promotes neurogenesis, survival, synaptogenesis, and plasticity. We hypothesize that if reduced MET signaling plays a role in AD pathogenesis, this might be reflected in the AD mouse models and as such provides opportunities for mechanistic studies on the role of HGF/MET in AD. Examining the 5XFAD mouse model revealed that MET protein exhibits age-dependent progressive reduction prior to overt neuronal pathology, which cannot be explained by indiscriminate loss of total synaptic proteins. In addition, genetic ablation of MET protein in cortical excitatory neurons exacerbates amyloid-related neuropathology in 5XFAD mice. We further found that HGF enhances prefrontal layer 5 neuron synaptic plasticity measured by long-term potentiation (LTP). However, the degree of LTP enhancement is significantly reduced in 5XFAD mice brain slices. Taken together, our study revealed that early reduction of HGF/MET signaling may contribute to the synaptic pathology observed in AD.

## Introduction

An estimated 50 million people worldwide are currently living with dementia (Scheltens et al., 2021), among which Alzheimer's disease (AD) accounts for the majority of these cases (Masters et al., 2015). AD is increasingly a global public health priority as the aging population increases. The current lack of effective therapeutics derives from our limited understanding of the mechanisms underlying AD pathogenesis, which hinders development of novel therapeutic and interventional approaches (Alexiou et al., 2019). Aside from the well-characterized extracellular amyloid plaques and neurofibrillary tangles composed of β-amyloid (Aβ) and phosphorylated tau proteins, an early pathology in AD is the loss of functional synapse and disruption in synaptic plasticity that occur before overt neurodegeneration (Cleary et al., 2005; Hsieh et al., 2006; Lacor et al., 2007; Shankar et al., 2007; Chen et al., 2010; Kim et al., 2014; Chung et al., 2016). Disruption of molecular mechanisms governing synaptic homeostasis and plasticity, such as activity-dependent long-term potentiation (LTP) (Gonzalez Burgos et al., 2012; Koch et al., 2012; Vanleeuwen and Penzes, 2012; Megill et al., 2015; Prieto et al., 2017), may render synapses dysfunctional and is considered an early pathological hallmark that instigates AD progression (Huh et al., 2016; Mango et al., 2019). While AD treatment strategies have been largely focused on Aβ and tau protein, the synapse itself could be a direct target to consider regarding disease intervention. Synaptic function may be preserved by preventing mechanisms of synapse degeneration and/or promoting mechanisms favoring homeostasis or regeneration (Smith et al., 2009).

Mesenchymal epithelial transition factor (MET) receptor tyrosine kinase and its ligand, hepatocyte growth factor (HGF), are expressed in the nervous system from prenatal development to adult life. The human *MET* gene plays a pleiotropic role in cell proliferation, morphogenesis, differentiation, survival, and regeneration of many tissue types. Upon HGF binding, MET is activated by autophosphorylation of intracellular tyrosine residues that serve as multi-substrate docking sites for signaling adaptors (Naldini et al., 1991a,b; Ponzetto et al., 1994). These adaptor proteins in turn activate pleiotropic molecular cascades including PI3K-AKT, MAPK/ERK, mTOR, and STAT (Stefan et al., 2001; Trusolino et al., 2010) to elicit a repertoire of cell behaviors collectively known as mitogenic, motogenic, and morphogenic. Our recent work reveals that MET in the developing cortical circuits controls dendritic spine formation and synaptogenesis (Qiu et al., 2011), refinement of circuit connectivity (Qiu et al., 2014; Peng et al., 2016), and the timing of excitatory synapse maturation (Qiu et al., 2011; Ising et al., 2019). Importantly, MET signaling persists in the adult brain but is restricted to the site of excitatory synapse (Akimoto et al., 2004; Eagleson et al., 2013) and is capable of modifying synaptic function and plasticity.

Existing literature supports that MET signaling is neurotrophic and neuroprotective in multiple neurodegenerative mouse models ranging from multiple sclerosis (Bai et al., 2012; Benkhoucha et al., 2014; Matsumoto et al., 2014), Parkinson's disease (Koike et al., 2006), to ALS (Genestine et al., 2011). HGF has also been shown to confer neuroprotection during stroke (Doeppner et al., 2011), ischemia (Shibuki et al., 2002), and Aβ-induced cognitive impairment in mice (Takeuchi et al., 2008). Moreover, the HGF-MET duo modifies central and peripheral immune functions (Benkhoucha et al., 2010), which are emerging as the key regulators of AD pathogenesis (Labzin et al., 2018; Ising et al., 2019; Gate et al., 2020). AD pathology accompanies numerous molecular changes that may coalesce into key signaling components, such as PI3K, STAT, PTEN, and mTOR (Oddo, 2012; Sanabria-Castro et al., 2017; Chen and Mobley, 2019; Yamazaki et al., 2019), which are also downstream players of MET signaling (Peng et al., 2013). Consistent with the posited neuroprotective role of MET signaling in AD, recent clinical literature demonstrated *reduced* levels of MET protein in the cerebral cortex and hippocampus of patients with AD (Hamasaki et al., 2014; Matsumoto et al., 2014). In addition, a transcriptome study also revealed *MET* as one of the major downregulated genes in AD brain (Liu et al., 2018). In this study, we further appraised the role of potential reduction of HGF/MET signaling in a 5XFAD AD mouse model. Our data support a novel contributory role of reduced HGF/MET signaling to the synaptic pathophysiology in this mouse model.

## Materials and Methods

### Animals and Disease Model

The 5XFAD mice used in this study were purchased from The Jackson Laboratory (Catalog #34848-JAX). This line overexpresses both mutant forms of human amyloid precursor protein gene *APP* (K670N/M671L, V717I, and I716V) and mutant forms of human *PS1* gene (M146L and L286V) under the Thy1 promoter (Oakley et al., 2006). Mice genotypes were identified according to JAX protocol. Mutant and wild-type alleles were amplified by polymerase chain reaction (PCR) using pairs of three primers (“wild type,” ACC TGC ATG TGA ACC CAG TAT TCT ATC; “common,” CTA CAG CCC CTC TCC AAG GTT TAT AG; and “mutant,” AAG CTA GCT GCA GTA ACG CCA TTT). All 5XFAD mice used in this study are heterozygotes. The forebrain-specific *Met* conditional knockout mice (cKO) were generated by breeding hemizygote male mice with a floxed *Met* gene (*Met*^fx/+^) and *emx*1^Cre^ knock-in allele (Gorski et al., 2002) to homozygous female *Met*^fx/fx^ mice (Judson et al., 2009; Qiu et al., 2014). Both *Met*^fx/fx^ and *emx*1^Cre^ lines were backcrossed onto the C57Bl/6 background and were genotyped by PCR as we previously reported (Xia et al., 2021). To obtain the *Met*^fx/fx^:*emx*1^cre^:5XFAD mice, *Met*^fx/+^:*emx*1^cre^:5XFAD mice were used as breeders. 5XFAD:*Met*^fx/fx^:*emx*1^cre^ mice (aka. 5XFAD:*Met*^cKO^) and their 5XFAD littermates (no *cre* transgene, irrespective of floxed *Met* allele status) were used for neuropathological comparisons. All experimental procedures conformed to NIH guidelines and were approved by the Institutional Animal Care and Use Committee of the University of Arizona.

### Immunohistochemistry / Immunofluorescence

Mice were euthanized with 3–5% isoflurane and transcardially cleared with cold 0.01 M PBS. Four percent paraformaldehyde (PFA) formulated in 0.1 M phosphate buffer (pH 7.4) was then perfused. Brains were dissected and postfixed in cold 4% PFA overnight. After cryoprotection in 30% sucrose for 2 days, brains were embedded in OCT cutting compound and sectioned on a sliding microtome (Leica SR-2000). About 40-μm-thick floating brain sections were washed 3X in 0.01 M PBS and permeabilized in PBS-0.2% Triton. For APP/Aβ+Iba1+Thio-S staining, the free-floating sections were blocked in primary antibody dilution solution (0.01M PBS containing 0.2% Triton, 5% normal goat serum, and 1% BSA) for 2 h. Anti-APP/Aβ primary antibody (clone 6E10, Biolegend, Cat# SIG-39320. Antibody Registry ID: AB_662798. 1:500 dilution) mixed with anti-Iba1 (#019-19741, Wako, 1:500 dilution) was applied for 24 h with slow rotation. Sections were washed 3X in 0.01 M PBS and further incubated with Alexa 555-conjugated goat anti-mouse antibody and Alexa 647-conjugated goat anti-rabbit antibody for 2 h. Sections were extensively washed in 0.01 M PBS and further stained with 0.025% Thio-S (prepared in 50% ethanol−50% PBS) for 10 min. After briefly destaining in 50% ethanol, sections were washed in 0.01 M PBS and mounted on the SuperFrost Plus slides (VWR Scientific, West Chester, PA) using DAPI-containing Vectashield mounting medium (H-1200, Vector Laboratories). Images were acquired on a LSM 710 confocal microscope (Zeiss GmbH, Germany) with appropriate laser lines and filters. We kept all the acquisition parameters constant through different experiments to allow comparisons, including photomultiplier tube detector gain, pinhole size, laser intensity, and image sizes. To count the number of Iba1+ microglia and coverage of APP/Aβ and Thio-S, we used maximum projection images from a 25-μm-Z stack plane (with 1-μm Z interval). Pseudo-colors of confocal image channels may be redefined to enhance visualization in figure preparation. To quantify signal area/size and co-localizations between channels, confocal .czi files were imported into Imaris or ImageJ for customized analyses.

### Western Blot Analysis

Western blots was performed using specific antibodies to detect proteins of interest. Total protein content of the micro-dissected PFC and CA1 tissues was quantified using a micro-BCA assay, after being homogenized in NP40 lysis buffer (FNN0021, Invitrogen) supplemented with proteinase inhibitor cocktail (Sigma-Aldrich, P8340, 1:100). The samples are then mixed with equal amount of 2 × Laemmli loading buffer and boiled for 5 min. Samples were separated by electrophoresis on 4–15% SDS-polyacrylamide gels. Proteins were then transferred to PVDF membrane (Immobilon-P, Sigma-Aldrich) and blocked with 0.01 M PBS-Tween 20 with 5% non-fat dry milk. Primary antibody was diluted in the same blocking solution and applied to the PVDF membrane in a humidified chamber on a glass plate covered with parafilm. After overnight primary antibody incubation, the PVDF membrane was washed three times in 0.01 M PBS-Tween 20. Secondary antibodies directed to the correct species that are conjugated to horseradish peroxidase were applied for 2 h. The protein signal was developed using an enhanced chemiluminescence method (SignalFire, Cell Signaling Technology) and captured on an X-ray film (Amersham ECL Hyperfilm). The following antibodies were used for this study: From Santa Cruz Biotechnology, mouse anti-MET (Cat# sc-8057); from Millipore/Chemicon, rabbit anti-GluA1 (AB5849), rabbit anti-PSD95 (AB9708); from Cell Signaling Technology, rabbit anti-phospho-MET (Y1234/1235) (#3077), rabbit anti-GAPDH (#5174). The final dilutions of antibodies were between 1:1,000 and 1:2,000. The optical intensity of protein signal band captured on Hyperfilm was digitized by a film scanner (Epson V850) into an 8-bit gray scale image and quantified by densitometry using ImageJ/FIJI.

### Synaptic Plasticity/Long-Term Potentiation

Standard extracellular field excitatory postsynaptic potential (fEPSP) recording was used to investigate long-term potentiation (LTP) changes in the prefrontal cortex (PFC) layer 5 (L5) region. Mice were euthanized using 3–5% isoflurane, followed by intra-cardiac perfusion of ice-cold choline solution (in mM: 110 choline chloride, 25 NaHCO_3_, 2.5 KCl, 1.25 NaH_2_PO_4_, 0.5 CaCl_2_, 7 MgSO_4_, 25-d glucose, 11.6 sodium ascorbate, and 3.1 sodium pyruvate, saturated with 95% O_2_/5% CO_2_) to improve brain slice viability. The brains were quickly harvested, and 350-μm-thick parasagittal slices were made on a vibratome (VT-1200S, Leica) at an angle that preserves intra-cortical L23>L5 connectivity (Qiu et al., 2011). Slices were cut in ice-cold choline solution, after which they were kept in artificial cerebrospinal fluid (ACSF, contains in mM: 126 NaCl, 2.5 KCl, 26 NaHCO_3_, 2 CaCl_2_, 2 MgCl_2_, 1.25 NaH_2_PO_4_, and 10-d glucose; saturated with 95% O_2_ /5% CO_2_) for 30 min at 35°C and then maintained at 24°C RT until recording.

Brain slices were transferred to an interface chamber (AutoMate Scientific) that is maintained at 32°C and superfused with ACSF saturated with 95% O_2_/5% CO_2_. This facilitates long-term slice viability. fEPSPs were recorded using a glass patch electrode in the PFC L5 region, while a bipolar tungsten stimulating electrode (FHC, Bowdoin, ME) was placed on the L23. The patch electrode had an electrical resistance of 1–2 MΩ at 1 kHz when filled with ACSF. Biphasic electrical stimuli were generated through a Digidata 1440A device (Molecular Devices, San Jose, CA) and delivered through an optic isolator (Iso-flex, A.M.P.I). Stimulus (100-μs duration) intensity ranged from 10 to 50 μA and was delivered at 0.05Hz for baseline and LTP recordings. fEPSP signals were amplified using a differential amplifier (model 1800, A–M Systems, Carlsborg, WA), low-pass filtered at 2 kHz, and digitized at 10 kHz through the Digidata 1440A board.

For each of the fEPSP recordings in PFC slices, a stimulus-response (input–output) curve was first obtained by measuring the fEPSP slope (first 1-ms response after fiber volley) as a function of the fiber volley amplitude. This curve was then used to quantify the strength of basal synaptic transmission. We adopted a stimulus intensity that produces a ~40–50% maximum monosynaptic fEPSP responses throughout the experiments. A 10-min stable baseline responses of stimulus-evoked fEPSPs were first obtained, and the paired-pulse responses at inter-pulse intervals ranging from 20 to 200 ms were recorded to assess potential changes in presynaptic transmission. Another pre-LTP 10-min baseline was then recorded, and LTP was induced by a theta-burst stimulation protocol, which consists of a 2-s long 5 Hz train (each train consists four pulses at 100 Hz) repeated 5 times at a 10-s interval (Qiu et al., 2006; Ma et al., 2019). fEPSP responses were recorded for an additional 1 h after LTP induction. Quantification of LTP amplitude was conducted in Clampfit 10.6, or using MATLAB by reading the .abf file with the *abf2load.m* function. To test the effects of HGF on LTP in control and 5XFAD slices, 10 nM recombinant human HGF (Millipore Sigma, Cat# GF116) was added to the ACSF perfusate for 30 min prior to the LTP induction. A subset of slices were collected after LTP studies, and L5 region was micro-dissected for western blot detection of phospho-MET (Tyr1234/1235), which was used as a surrogate of MET activation.

### ELISA Measurement of Aβ1-42 Levels

Brains were weighed, sliced, and subjected to sequential Aβ extraction. Prefrontal cortical tissues (L5 region) were dissected and homogenized in 2% SDS-RIPA buffer (containing: 150 mM NaCl, 1% NP-40, 50 mM Tris-base, 2% SDS in aqueous solution, 5 mM EDTA, and 0.5% Na-deoxycholate). The SDS-RIPA buffer also contains protease inhibitor cocktail (1:100, Sigma P8340). Tissue homogenates were incubated on ice for 15 min to extract proteins, followed by centrifugation at 16,000 g for 90 min at 4 C. The supernatant containing RIPA-soluble fraction of Aβ_1−42_ was collected. The pellet containing the insoluble fraction was further incubated for 30 min with 15X volume 70% fomic acid at room temperature and further centrifuged at 16,000 g for 60 min at 4°C. The supernatant collected now contains the RIPA-insoluble fractions of Aβ_1−42_. For both soluble and insoluble fractions, total protein content was determined using the micro-BCA method. Aβ_1−42_ levels were measured using an amyloid beta 42 mouse colorimetric ELISA Kit (Cat# KMB3441, Thermo Fisher) that is provided in a 96-well format, according to the manufacturers' instructions. A microplate reader (Tecan) was used to measure absorption at 450 nm.

### Statistical Analyses

The experimenters were blinded to grouping/genotype information during data collection and analyses. Sample sizes and number of independent experiments were estimated by power analyses using an R script (“pwr” package on CRAN) that takes pre-specified effect size, type I and II errors as input arguments. Shapiro–Wilk test and *F* test were first employed to test normality and equal variance. All data that passed normality/equal variance tests were reported as mean ± SEM (standard error of the mean). For normal-distributed/equal variance data, Student's *t*-test or multiple *t*-tests were used. Statistical analyses and graphing were performed using GraphPad Prism 8.0, Microsoft Excel, and MATLAB. *P-*Value < 0.05 was considered statistically significant for all tests. Figures were prepared using Adobe Creative Cloud.

## Results

### 5xFAD Mice Show Age-Dependent Decrease of MET Protein in Prefrontal Cortex and Hippocampus

**Figure 1:**
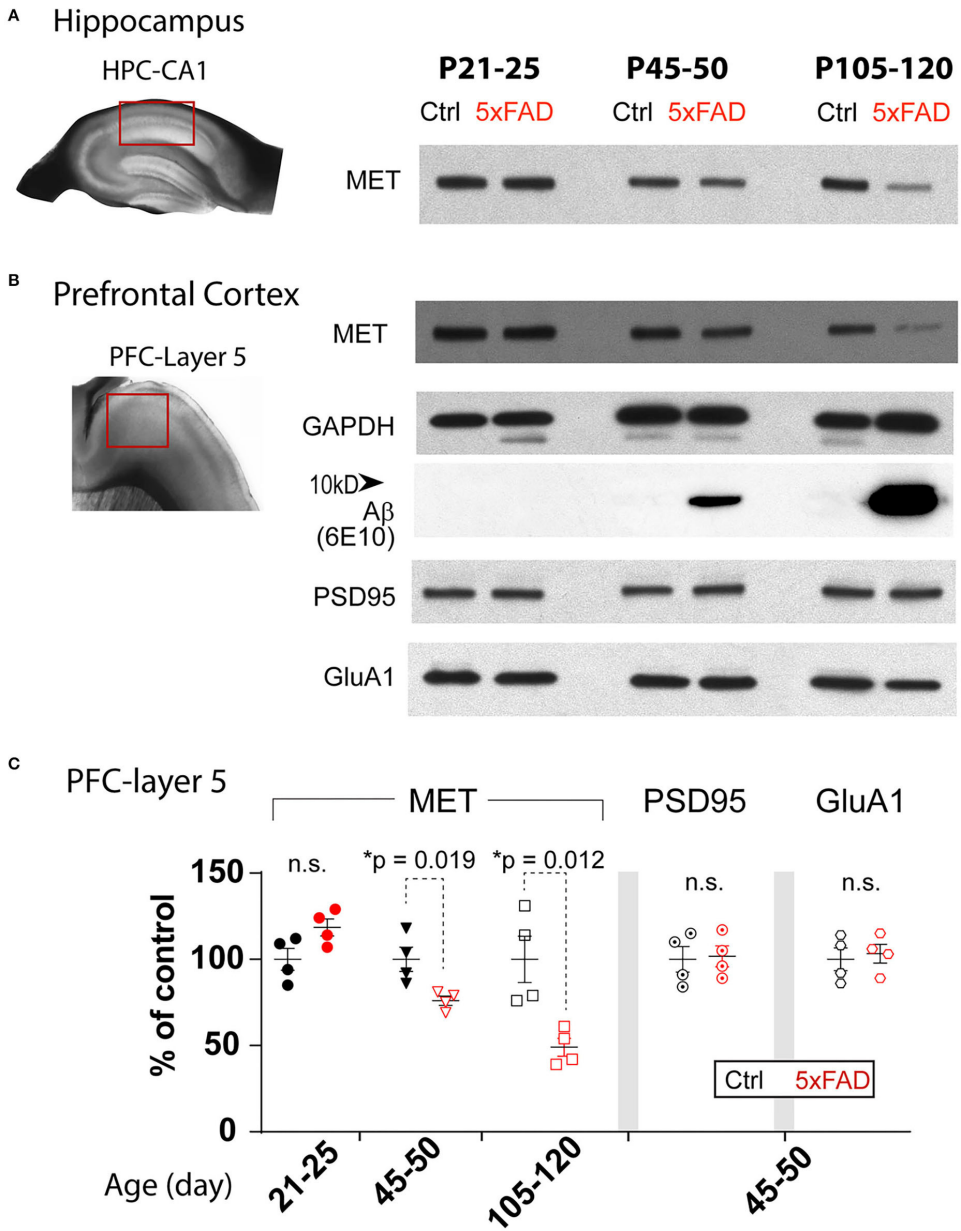
Age-dependent decrease of MET protein in PFC and HPC in 5XFAD mice. **(A)** Western blot showing MET protein levels from control and 5XFAD hippocampus CA1 regions at three ages: P21-25, P45-50, and P105-120. **(B)** MET and other synaptic protein levels in micro-dissected L5 PFC tissues. The same age-dependent reduction of MET protein is observed (signals normalized to GAPDH loading control). Abrupt increase in Aβ levels was seen in P45-50 and older tissues. Other synaptic proteins, including PSD95 and GluA1, were not different between control and 5XFAD PFC L5 tissues. **(C)** Quantification of western blot results in PFC tissues. MET protein shows a significant reduction at P45-50 (*p = 0.019) and P105-120 (*p = 0.012). No difference in the levels of PSD95 and GluA1 was seen.

It has been reported that AD brain shows early reduction of MET (Hamasaki et al., 2014; Matsumoto et al., 2014). We examined MET protein levels in the heterozygote 5xFAD mice PFC- L5 and HPC-CA1 tissues at three different ages (Figure 1): P21-25, during which the brain shows no overt Aβ/amyloid pathology; P45-50, a pre-symptomatic stage when Aβ is dramatically increased; and P105-120, during which amyloid plaques are prominent (Oakley et al., 2006). Western blot analyses revealed that compared to littermate controls, MET proteins in 5xFAD tissues were of similar levels at P21-25 in both PFC-L5 and HPC-CA1 tissues (Figures 1A,B), but were dramatically reduced at P45-50 in both regions (Figures 1A,C, PFC-L5, *p* = 0.019, *n* = 4; HPC-CA1, *n* = 4, quantification not shown). At P105-120, MET is severely depleted in both brain regions in 5XFAD brain (Figures 1A,C, PFC-L5, *p* = 0.012, *n* = 4; HPC-CA1, *n* = 4, quantification not shown). MET protein reduction in PFC and HPC parallels the abrupt, qualitative increase in Aβ load in the 5XFAD mice at this age (Figure 1B).

The early reduction of MET proteins in 5XFAD brain can be due to specific reduction of HGF/MET signaling that is related to AD pathology or APP/Aβ overloading, or it could be simply a non-specific effects of early synapse loss. We therefore probed levels of PSD95, a postsynaptic protein, and GluA1, an AMPA receptor subunit of the excitatory synapse, in the PFC-L5 tissues. After normalizing to the GAPDH loading controls, quantification of MET, PSD95, and GluA1 is shown in Figure 1C. We found that PSD95 and GluA1 protein levels were not changed in PFC at all three ages (Figure 1C, *p* > 0.05 for quantification of PSD95 and GluA1 at P45-50, *n* = 4; other ages, data not shown). As such, the early reduction of MET protein in PFC and HPC at P45-50 or later cannot be explained by the gross loss of synapse at this stage, as other synaptic proteins markers were not affected.

### Genetic Ablation of MET Signaling Exacerbates Aβ-Related Neuropathology in 5XFAD Mice

**Figure 2:**
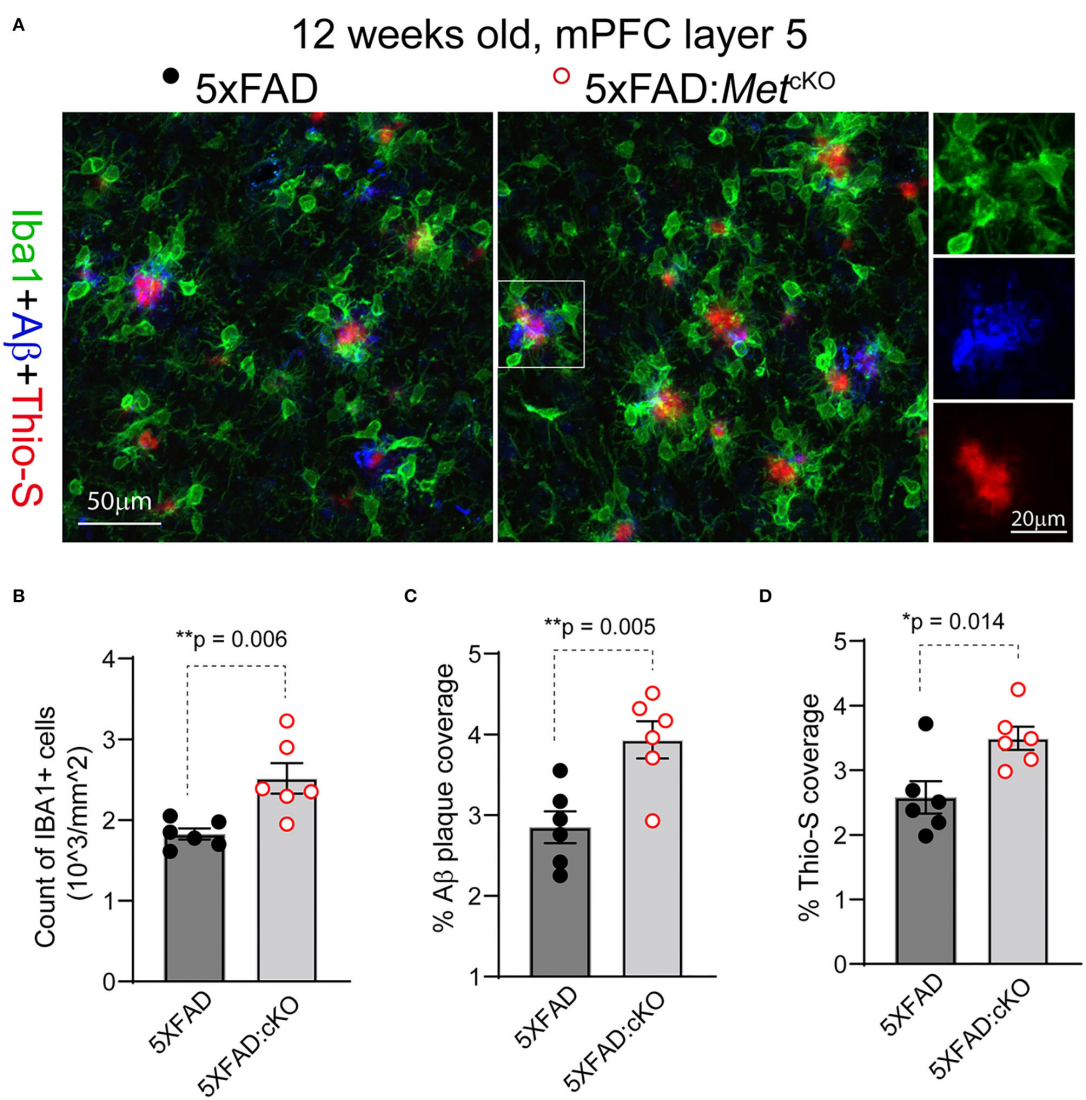
Genetic ablation of MET signaling exacerbates Aβ-related neuropathology. **(A)** Representative confocal images showing triple staining of Iba1/APP-Aβ/Thio-S in 12-week-old 5XFAD and 5XFAD:*Met*^cKO^ mice mPFC L5 regions. **(B)** Quantification of density of Iba1+ active microglia. 5XFAD:*Met*^cKO^ mice show significantly increased microglia density (**p = 0.006). **(C)** Quantification of density of APP/Aβ+ plaque area. 5XFAD:*Met*^cKO^ mice show significantly increased plaque coverage (**p = 0.005). **(D)** Quantification of Thio-S+ percentage areas. Increased Thio-S+ coverage was seen in 5XFAD:*Met*^cKO^ mice (*p = 0.014).

The early reduction in MET protein in 5XFAD mice, together with reported neuroprotective role of HGF/MET in animal models (Doeppner et al., 2011), suggests that MET could be a protective factor in AD. If this is the case, ablation of MET signaling, which could be achieved through the cre-loxP technology, may aggravate neuropathology in the 5XFAD mice. We crossed the forebrain excitatory neuron-specific *Met* cKO mice (*Met*^fx/fx^:*emx*1^cre^) to the 5XFAD mice (refer to Methods). *Emx*1^cre^ drives inactivation of the *Met* gene from mid-gestation stage. We compared the 5XFAD mice (no *cre* transgene, irrespective of floxed *Met* allele status) with their littermate mice harboring the inactivated *Met* gene (5XFAD:*Met*^cKO^) for their neuropathological markers. We immunostained PFC sections for Iba1, a marker for reactive microglia; APP/Aβ, and Thio-S, a marker for non-soluble fibrillary β-sheet forms of amyloid plaques (Figure 2A).

We first counted the number and density of Iba1+ microglia from L5 mPFC regions in 12-week-old (postnatal days 82–89) 5XFAD and the 5XFAD:*Met*^cKO^ mice. 5XFAD:*Met*^cKO^ mice show significantly increased Iba1+ microglia density [Figure 2B, number of cells/mm^2^: 5XFAD, 1827 ± 67; 5XFAD:*Met*^cKO^, 2518 ± 188. *t*_(10)_ = 2.43, *p* = 0.006]. Next, we quantified the APP/Aβ+ plaque coverage as a percentage of the imaged L5 area. 5XFAD:*Met*^cKO^ mice show significantly increased percentage areal coverage of APP/Aβ [Figure 2C, 5XFAD, 2.85 ± 0.19; 5XFAD:*Met*^cKO^, 3.93 ± 0.23. *t*_(10)_ = 3.58*, p* = 0.005]. To estimate the density of non-soluble fibrillary forms of amyloid plaques, we quantified the Thio-S+ areas. 5XFAD:*Met*^cKO^ mice also show significantly increased percentage areal coverage of Thio-S staining [Figure 2D, 5XFAD, 2.58 ± 0.25; 5XFAD:*Met*^cKO^, 3.49 ± 0.18. *t*_(10)_ = 2.98, *p* = 0.014]. Taken together, these data show that genetic ablation of MET signaling developmentally aggravates or accelerates neuropathology in the 5XFAD mouse model.

### Genetic Ablation of MET Signaling Increases Production of Aβ in 5XFAD Mice

**Figure 3:**
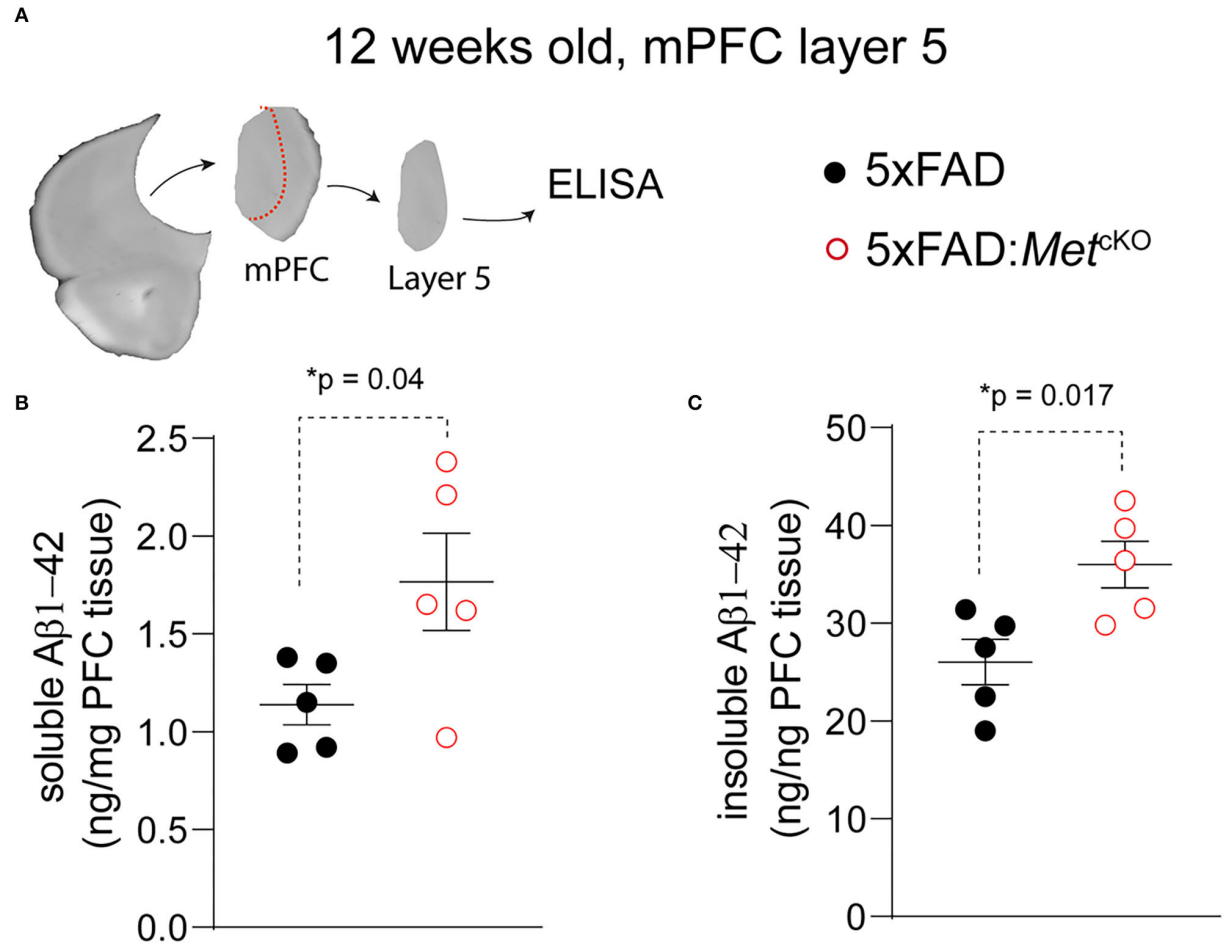
Genetic ablation of MET signaling increases Aβ production in 5XFAD mice. **(A)** Schematic illustration of micro-dissected mPFC L5 tissues that are used for ELISA measurement of Aβ_1−42_. **(B)** 5XFAD:*Met*^cKO^ mice L5 mPFC tissues show significantly increased soluble forms of Aβ_1−42_ (*p = 0.04). **(C)** Compared to 5XFAD tissues, 5XFAD:*Met*^cKO^ mice L5 mPFC tissue shows significantly increased insoluble forms of Aβ_1−42_ (*p = 0.017).

Based on the observed changes in neuropathology, we asked whether *Met* cKO may exacerbate production of Aβ, which is an essential component of the phenotypic signature of AD. We again used the ~12-week-old mPFC tissues and micro-dissected the L5 regions (Figure 3A). We biochemically isolated the 2% SDS soluble and insoluble forms of beta-amyloid and used an ELISA kit to measure the Aβ_1−42_ species (refer to Methods). We found that *Met* cKO significantly increased the levels of soluble Aβ_1−42_ [Figure 3B, 5XFAD, 1.14 ± 0.10 ng/mg total protein; 5XFAD:*Met*^cKO^, 1.77 ± 0.25. *t*_(8)_ = 2.33, *p* = 0.04]. In addition, the amyloid plaque associated insoluble fraction of Aβ_1−42_ was even more dramatically elevated [Figure 3C, 5XFAD, 26.0 ± 2.31 ng/mg total protein; 5XFAD:*Met*^cKO^, 35.9 ± 2.39. *t*_(8)_ = 2.99, *p* = 0.017]. These data are consistent with the neuropathological findings and suggest that MET signaling could be a neuroprotective factor during AD pathogenesis.

#### Differential Responses to HGF-Induced Plasticity in the PFC L5 Synapses in 5XFAD Mice

**Figure 4:**
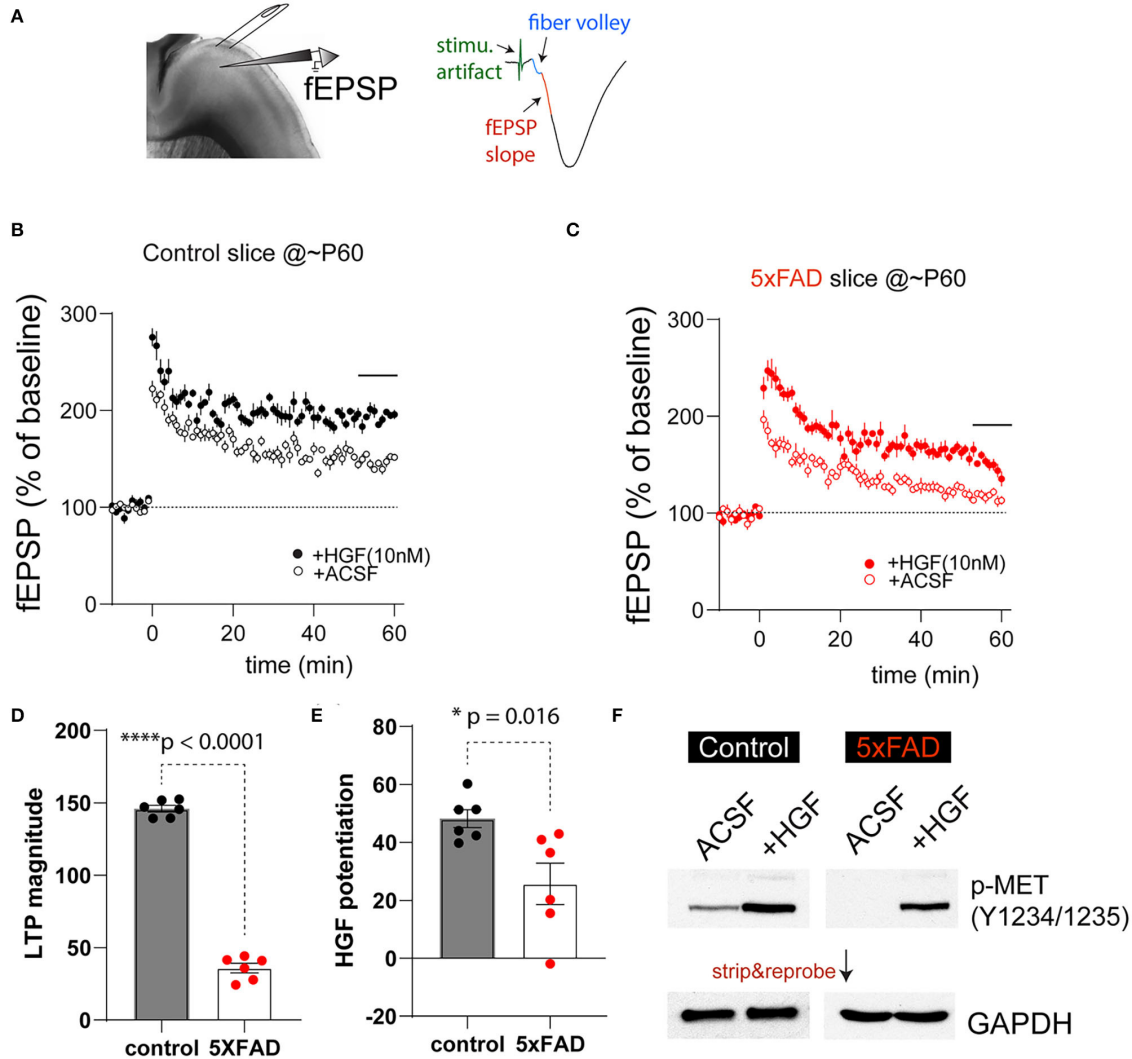
5XFAD mice show impaired PFC-L5 synaptic plasticity and HGF-induced LTP enhancement. **(A)** Schematic illustration of LTP recording in PFC-L5. A bipolar stimulating electrode was placed in L23 to elicit monosynaptic fEPSP responses (*right*). The first 1-ms fEPSP response after fiber volley was used to calculate slope of response. **(B)** LTP time course in control slices in the absence (ACSF) and the presence of 10 nM HGF. HGF clearly elevates LTP magnitude. **(C)** LTP time course in 5XFAD slices in the absence (ACSF) and the presence of 10 nM HGF. HGF shows smaller enhancement of LTP magnitude in 5XFAD slices compared to that from control slices. **(D)** Quantification of theta-burst-induced LTP magnitude in control and 5XFAD slices (last 6-min recordings were used). LTP magnitude in 5XFAD slices was dramatically reduced compared to that from control slices (****p < 0.0001). **(E)** Quantification of HGF-induced potentiation of the LTP magnitude. 5XFAD slices show significantly reduced HGF-induced LTP enhancement compared to that from control slices (*p = 0.016). **(F)** Differential basal levels of p-MET (Y1234/1235) and HGF-induced p-MET levels in 5XFAD vs. control slices that were collected after LTP recordings. *N* = 4, quantification not shown.

It has been previously shown that HGF, when acutely applied to juvenile rat hippocampus slices, enhanced synaptic LTP in the HPC-CA1 region. This enhancement is most likely due to augmenting NMDA receptor-mediated currents in slices by HGF (Akimoto et al., 2004). Our recent work also suggests the timing of HGF/MET signaling affects the age-dependent synaptic plasticity in the hippocampus (Ma et al., 2019). We next investigated how synaptic plasticity in 5XFAD and control PFC slices response acutely to HGF application, and any potential differences may be due to the differential MET signaling state. We chose to study the plasticity measures in ~2-month (P55-68)-old 5XFAD mice and littermate controls by conducting fEPSP recording at the PFC L23>L5 synapse. After obtaining stable baseline fEPSP responses, LTP was induced by theta-burst stimulation (Figure 4A). It was found that in control slices, theta-burst stimulation on its own leads to robust LTP that lasts at least 1 h postinduction (Figure 4B). This LTP magnitude was dramatically elevated when HGF (10 nM) was pre-applied for 30 min (Figure 4B). In comparison, LTP magnitude induced by theta-burst alone was smaller in 5XFAD PFC slices, although HGF was capable of leading to an enhancement (Figure 4C). When LTP magnitude was quantified using the last 6-min recording, it was found that theta-burst-induced LTP magnitude was significantly reduced in 5XFAD slices [Figure 4D, 5XFAD, 35.8 ± 3.3%; control, 145.9 ± 2.3%. *t*_(10)_ = 27.2, *p* < 0.0001]. We also quantified HGF-induced LTP enhancement. HGF leads to significantly lower LTP enhancement in 5XFAD slices [Figure 4E, Control, 41.2 ± 3.1; 5XFAD, 25.7 ± 7.2. *t*_(10)_ = 2.88, *p* = 0.016].

The reduced level of theta-burst-induced LTP and HGF enhancement of LTP magnitude in 5XFAD PFC slices may reflect the overall lower levels of MET at this age (Figures 1B,C) or reduced baseline MET signaling levels at this age. We next quantified p-MET (Tyr1234/1235) levels in micro-dissected L5 tissues after recording under the above four experimental conditions. Strikingly, we found that p-MET levels in 5XFAD slices were very low, so that it is practically undetectable in 5XFAD slices under ACSF condition (Figure 4F). In comparison, p-MET is clearly detectable in control littermate slices. More importantly, 10 nM HGF application, which results in enhanced LTP (Figures 4B,C), also leads to quantitatively or qualitatively increased p-MET levels in control and 5XFAD slices (Figure 4F, *N* = 4 biological replicates, quantification now shown). These data suggest that baseline activation of MET is much lower in 5XFAD mouse brain at a pre-symptomatic age, yet the endogenous level of MET is still capable of responding to HGF-induced signaling.

## Discussion

In this study, we found early reduction of MET receptor tyrosine kinase in the 5XFAD mouse model for AD. This is consistent with clinical literature reporting a reduction of MET protein in the AD brain, including the hippocampus and cortical regions (Hamasaki et al., 2014; Matsumoto et al., 2014; Liu et al., 2018). Importantly, early MET reduction in the 5XFAD mice cannot be due to overall early loss of synapse, as other synaptic proteins (PSD95, GluA1) were not changed at early ages. It is also likely the reduction of MET signaling promotes AD progression and severity, as genetic ablation of *Met* results in exacerbated pathological changes in the 5XFAD mice. These are reflected by the increased amyloid plaque deposition, dense core fibrillar forms of plaque, increased soluble and insoluble forms of Aβ, and increased microglia activation. All of these observations suggest that HGF/MET reduction may contribute to AD pathogenesis in this mouse model featuring aggressive amyloid deposition.

Our findings have translational implications for AD therapeutics. The pathophysiology of AD is extremely complex. Existing literature suggests that protein aggregation, neuroinflammation, disrupted energy metabolism, vascular pathology, and immune dysregulation all play a role (Kinney et al., 2018; Guo et al., 2020; Yu et al., 2021). HGF/MET signaling is also pleiotropic, engaging a plethora of molecular pathways that are reportedly disrupted in AD (Wright and Harding, 2015; Desole et al., 2021). At cellular level, early synaptic loss and impairment of plasticity occur prior to overt neuronal loss and degeneration (Oakley et al., 2006; Richard et al., 2015; Forner et al., 2021; Oblak et al., 2021), which may instigate progressive decline in memory and cognition. As such, focusing on the synaptic regeneration and preserving plasticity across the lifespan presents some important conceptual and strategic issues regarding translational AD research, may inform future clinical practice with the aim to preserve synapse and circuit connectivity, and restore cognitive function in AD and other neurodegenerative diseases.

The HGF/MET signaling is highly pleiotropic and influences a plethora of early neurodevelopmental events, including neural induction (Streit et al., 1995), proliferation (Ieraci et al., 2002), neurite outgrowth (Maina et al., 1997, 1998; Korhonen et al., 2000; Gutierrez et al., 2004), and survival and regeneration (Hamanoue et al., 1996; Wong et al., 1997; Davey et al., 2000; Maina et al., 2001). We and others have shown that MET is a temporally- and spatially-regulated receptor enriched in dorsal pallial-derived structures during mouse forebrain development. Peak levels of MET expression in cortex coincide with periods of rapid neuronal growth and synaptogenesis. MET in developing cortical circuits controls dendritic spine formation and synaptogenesis (Qiu et al., 2011), refinement of circuit connectivity (Qiu et al., 2014; Peng et al., 2016), and the timing of excitatory synapse maturation (Qiu et al., 2011; Ising et al., 2019). The cellular and circuit origins of HGF, on the other hand, are less understood. Early immunohistochemistry staining suggests astrocytes and a small number of microglia may be the main source of HGF (Yamada et al., 1994, 1997; Yamagata et al., 1995). It is thus likely that the HGF/MET signaling duo involves multiple cellular types and may be modified by physiology states of the brain. In contrast to its neurodevelopmental role, MET signaling in adult brain is less understood. However, MET protein persists in adult brain but is restricted to the site of excitatory synapse (Eagleson et al., 2013) and is capable of modifying synaptic function and plasticity (Akimoto et al., 2004). We recently found that in *Met* conditional knockout (cKO, *Met*^fx/fx^:*emx*1^cre^) mice, there was a disruption in hippocampus LTP and an early cognitive decline (Ma et al., 2019); conversely, in transgenic mice overexpressing *Met*, cortical excitatory neurons exhibit altered synaptic proteins and the timing of critical period plasticity (Chen et al., 2020). MET signaling also engages molecular mechanisms governing *de novo* spine morphogenesis; for example, MET signaling activates small GTPases (Cdc42, Rac1) to control actin dynamics (e.g., cofilin phosphorylation). The signaling is capable of promoting dendritic spines/synapses morphogenesis *de novo* in response to neuronal activity (Qiu et al., 2014; Chen et al., 2020). Therefore, existing literature suggests that MET signaling is neuroprotective, modifies synaptic function and plasticity, and has synapse regeneration potential.

In summary, our study revealed that MET protein and its mediated signaling are reduced in the 5XFAD mouse model for AD. The study complements recent clinical literature reporting reduced MET protein levels in AD patient's brain and the posited beneficial effects of HGF/MET activation in AD therapeutics (Sharma, 2010; Hua et al., 2022) and further supports the view that HGF/MET signaling is neurotrophic and neuroprotective. The current findings, combined with our recent work in developmental neurobiology that revealed molecular mechanisms that controls *de novo* spine genesis (Qiu et al., 2014; Peng et al., 2016; Chen et al., 2020), suggest that restoring/enhancing MET signaling levels may represent a promising direction in AD therapeutics.

## Data Availability Statement

The original contributions presented in the study are included in the article/supplementary material, further inquiries can be directed to the corresponding author/s.

## Ethics Statement

The animal study was reviewed and approved by Institutional Animal Care and Use Committee of the University of Arizona.

## Author Contributions

JW and XM performed the experiments. AN and YC conducted partial data analyses. LZ performed animal husbandry and genotyping. YC performed coded data analysis in MATLAB. SQ designed and supervised the study and acquired funding. All authors contributed to the article and approved the submitted version.

## Funding

This work was supported by institutional startup funding from the University of Arizona (SQ).

## Conflict of Interest

The authors declare that the research was conducted in the absence of any commercial or financial relationships that could be construed as a potential conflict of interest.

## Publisher's Note

All claims expressed in this article are solely those of the authors and do not necessarily represent those of their affiliated organizations, or those of the publisher, the editors and the reviewers. Any product that may be evaluated in this article, or claim that may be made by its manufacturer, is not guaranteed or endorsed by the publisher.
